# Renal function at the time of a myocardial infarction maintains prognostic value for more than 10 years

**DOI:** 10.1186/1471-2261-11-37

**Published:** 2011-06-27

**Authors:** Thomas Kümler, Gunnar H Gislason, Lars Kober, Finn Gustafsson, Morten Schou, Christian Torp-Pedersen

**Affiliations:** 1 Dept. of Cardiology, Rigshospitalet, Copenhagen University Hospital, Denmark; 2 Dept. of Cardiology, Gentofte University Hospital, Copenhagen, Denmark; 3 Dept. of Cardiology, Hilleroed University Hospital, Hilleroed, Denmark

## Abstract

**Background:**

Renal function is an important predictor of mortality in patients with myocardial infarction (MI), but changes in the impact over time have not been well described.

We examined the importance of renal function by estimated GFR (eGFR) and se-creatinine as an independent long-term prognostic factor.

**Methods:**

Prospective follow-up of 6653 consecutive MI patients screened for entry in the Trandolapril Cardiac Evaluation (TRACE) study. The patients were analysed by Kaplan-Meier survival analysis, landmark analysis and Cox proportional hazard models. Outcome measure was all-cause mortality.

**Results:**

An eGFR below 60 ml per minute per 1.73 m^2^, consistent with chronic renal disease, was present in 42% of the patients. We divided the patients into 4 groups according to eGFR. Overall, Cox proportional-hazards models showed that eGFR was a significant prognostic factor in the two groups with the lowest eGFR, hazard ratio 1,72 (confidence interval (CI) 1,56-1,91) in the group with the lowest eGFR. Using the eGFR group with normal renal function as reference, we observed an incremental rise in hazard ratio. We divided the follow-up period in 2-year intervals. Landmark analysis showed that eGFR at the time of screening continued to show prognostic effect until 16 years of follow-up. By multivariable Cox regression analysis, the prognostic effect of eGFR persisted for 12 years and of se-creatinine for 10 years. When comparing the lowest group of eGFR with the group with normal eGFR, prognostic significance was present in the entire period of follow-up with a hazard ratio between 1,97 (CI 1,65-2,35) and 1,35 (CI 0,99-1,84) in the 2-year periods.

**Conclusions:**

One estimate of renal function is a strong and independent long-term prognostic factor for 10-12 years following a MI.

## Background

The prevalence of cardiovascular disease rises with declining renal function [1-4]. The significance of impairment of kidney function for cardiovascular outcome after myocardial infarction (MI) has been examined in patients with heart failure [5], impaired left ventricular function [6] and in patients undergoing coronary artery bypass grafting (CABG) [7].

Several studies have examined cardiovascular outcomes in patients with end-stage renal disease but data on the risk in patients with lesser degrees of renal dysfunction and MI is lacking [8-11]. Moreover, several studies are limited by a short follow-up period and by relying on serum creatinine (se-creatinine) level, which is considered a suboptimal indicator of renal function. Long-term prognosis after myocardial infarction has generally improved over the last decades [12] and consequently it is of interest to study not just markers of short- or intermediate term prognosis but rather predictors of long-term - i.e. more than 10 years - prognosis. In fact, no information on the influence of estimates of renal function on such long-term prognosis after myocardial infarction is available.

We undertook a retrospective study of 6676 patients admitted with myocardial infarction and who were screened for entry in the Trandolapril Cardiac Evaluation Registry (TRACE) study. Renal function was evaluated by estimated GFR and s-creatinine and patients were followed for up to 17 years to systematically evaluate the importance of renal function as an independent prognostic factor evaluated at the time of the index infarction.

## Methods

### Subject and study design

The Trandolapril Cardiac Evaluation Registry (TRACE) registry has been described in detail previously [13,14]. In brief, the TRACE registry consist of the 6676 MI patients screened for entry in the TRACE study, which was a double-blind, randomised, parallel group, placebo-controlled study of trandolapril versus placebo in patients with left ventricular dysfunction after MI. The study was conducted in 27 centres in Denmark and included consecutive patients admitted with MI. Participating centres recorded complications on a daily basis, and performed an echocardiography 2-6 days after the infarction. The worst status for each complication was recorded in the register. The diagnostic criteria of myocardial infarction were chest pain and/or electrocardiographic changes suggestive of ischemia or infarction, accompanied by elevated cardiac enzymes. Left ventricular systolic function was evaluated in a core lab as wall motion index using a 9-segment model and a reverse scoring system. Wall motion index multiplied by 30 approximates left ventricular ejection fraction, hence a wall-motion index of 1.2 corresponds to an ejection fraction of 35%. The technique has previously been described in detail and validated [15]. Of the screened patients, 1749 (26.2%) were randomised to trandolapril or placebo in the TRACE study.

The TRACE study was approved by all regional ethical committees in Denmark and complies with the Declaration of Helsinki. The study was registered with the National Board of Health and the Danish Data Protection Agency. All participating patients provided informed consent.

### Estimation of renal function

Se-creatinine was recorded as the first measurement performed at admission in all patients, and given the half-life of se-creatinine the measurement represents kidney function prior to the MI in the vast majority of the patients. Direct measurement of GFR is often limited by practical problems, especially in acutely ill patients. Of several reliable equations, we estimated GFR (eGFR) with the use of the four-component MDRD equation incorporating age, race, sex and serum creatinine level [16]:

For women the product of this equation was multiplied by a correction factor of 0.742. We did not use a correction factor for race because all included patients were Caucasian.

We divided eGFR into four categories (less than 45.0 (eGFRgroup 4), greater than or equal to 45.0 and less than 60.0 (eGFRgroup 3), greater than or equal to 60.0 and less than 75.0 (eGFRgroup 2) and greater than or equal to 75.0 (eGFRgroup 1) ml per minute per 1.73 m^2^), incorporating the guidelines of the National Kidney Foundation [17].

In order to compare the prognostic significance of creatinine and eGFR, we divided se-creatinine into 4 categories (se-creatinine less or equal to 60 (cregroup 1), greater than 60 and less than 75 (cregroup 2), greater than 120 and less than 180 (cregroup 3), greater than 180 (cregroup 4) μmol/l).

### Follow-up data

All Danish citizens are given a unique and permanent personal identification number that allows cross-linkage between registries. All deaths in Denmark are registered in the central person registry within 2 weeks and all deaths are confirmed by a death certificate. Follow-up mortality data were provided by a computerized analysis from the Danish Central Personal Registry by 16.06.2008.

### Statistical analysis

eGFR is presented in categories for descriptive purposes but was used as a continuous variable in statistical tests. The baseline characteristics of the study population were compared with a t-test for continuous variables and a chi-square test for discrete variables. Mortality was analysed with Kaplan-Meier curves. We used Landmark analyses to illustrate the prognostic significance of renal function in 2-year intervals. All-cause mortality was compared with multivariable Cox proportional-hazards regression models. We used stepwise models including increasing number of variables. Model 1 is a univariate analysis, model 2 included age and gender and model 3 in addition included all covariates (Body mass index, previous AMI, angina pectoris, congestive heart failure, diabetes mellitus, wall motion index, systemic hypertension, thrombolytic therapy). In all models either se-creatinine group or eGFR were included in the model. The model assumptions (linearity of continuous variables, proportional hazards assumption and lack of interaction) were fulfilled. All p-values were two-sided, and a P value of less than 0.05 was considered statistical significant.

Statistical analyses were performed with the Statistical Analysis System software package, version 9.1 (SAS Institute, Cary, NC, USA).

## Results

A total of 7001 MI's in 6676 patients were reported from May 1990 to August 1992. By the end of follow-up, death was reported in 5231 patients (78.4%). A total of 38 non-Danish patients (0.56%) were censored at the day of hospital discharge. There were missing data on renal function in 23 patients, who were excluded from our analyses.

Baseline characteristics of the 6653 patients included in our study are presented in table 1. Forty-two% had an estimated GFR below the suggested value for chronic kidney disease (<60 ml per minute per 1.73 m^2^). With declining renal function patients were older, had more co-morbidity, were less often treated with thrombolytic therapy but more often with diuretics, had worse left ventricular systolic function and were in a more severe clinical state evaluated by New York Heart Association (NYHA) and Killip class. There were few patients with recurrent MI (N = 198, 3,72%) or pulmonary embolism (N = 27, 0,27%). Only 178 patients (3,33%) were receiving antiarrhytmic therapy at discharge.

**Table 1:** Patient characteristics according to classification of renal function by eGFR.

	eGFR group 1 **eGFR ≥ 75.0 ml per minute per 1.73 m**^**2**^ (n = 1772)	eGFR group **2 60.0 ≤ ≤74.9 ml per minute per 1.73 m**^**2**^ (n = 2071)	eGFR group 3 **45 ≤ ≤59.9 ml per minute per 1.73 m**^**2**^ (n = 1726)	eGFR group 4 **eGFR <45 ml per minute per 1.73 m**^**2**^ (n = 1107)	P Value*
Age (SD)	59.7	66.6	71.1	75.0	<0.0001
Gender,%					
Women	16.9	28.4	42.5	49.9	<0.0001
Men	83.1	71.6	57.5	50.1	
Body mass index (SD)	25,8	25.9	25.6	25.2	<0.0001
Creatinine (mean), μmol/l	80.1	95.6	112.1	174.5	<0.0001
Hypertension,%	15.9	20.4	25.8	32.8	<0.0001
Diabetes,%	6.7	9.8	12.1	17.2	<0.0001
Angina pectoris%	27.5	36.0	42.1	45.2	<0.0001
Previous MI,%	17.0	22.6	26.0	30.8	<0.0001
Heart failure,%	34.2	48.8	64.1	77.3	<0.0001
Smoking,%					
Previously	16.9	22.2	24.9	25.1	<0.0001
Currently	65.8	52.9	44.5	36.2	
Thrombolysis,%	49.7	45.3	37.4	23.7	<0.0001
Previous stroke,%	4.6	7.0	9.4	14.2	<0.0001
Diuretic treatment,%***	25.2	40.2	53.7	70.0	<0.0001
Digoxin at discharge,%***	7.2	13.9	21.0	31.3	<0.0001
ACE inhibitor at discharge,%***	4.2	5.8	9.8	15.3	<0.0001
NYHA,%					
Class I	68.3	62.2	51.6	38.9	<0.0001
Class II	25.6	27.6	31.4	33.4	
Class III	2.2	3.8	5.4	6.6	
Class IV	2.6	4.3	7.8	15.5	
Killip**,%					
Class I	88.6	82.8	74.2	62.8	<0.0001
Class II	9.1	13.5	18.6	21.9	
Class III	0.5	1.3	2.6	5.4	
Class IV	1.9	2.3	4.7	9.9	
Wall motion index,%					
>1,6	42.5	33.4	28.9	21.8	<0.0001
1,3-1,6	24.7	24.5	22.4	21.8	
0,8-1,2	26.2	33.1	35.4	34.2	
<0,8	2.0	4.0	6.3	9.8	
Not classified	4.5	5.0	7.0	12.6	
Ventricular fibrillation.%****	5.4	6.5	9.3	8.0	<0.0001
Ventricular tachycardia,%****	12.2	12.8	13.1	12.6	0.8744
Atrial fibrillation,%****	12.3	18.2	25.6	32.4	<0.0001

*for difference between groups.

** Worst Killip class during index hospitalization

SD, standard deviation; NYHA, New York Heart Association;

P-values calculated with the use of chi^2^-test for discrete variables and t-test for continuous variables.

***At discharge.

****During hospital stay (day 5 to discharge)

**Figure 1:**
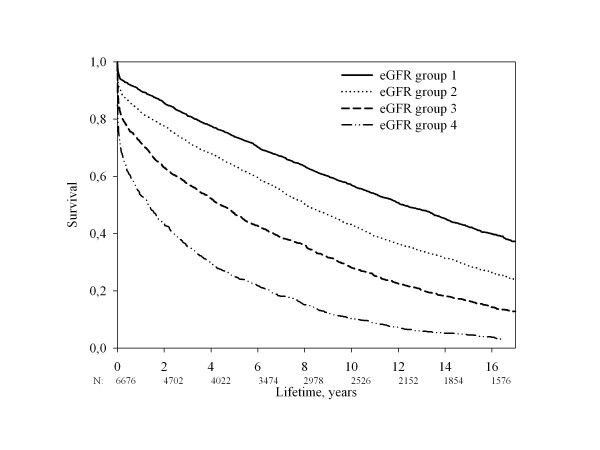
**Unadjusted all-cause mortality stratified by eGFR group**.

**Figure 2:**
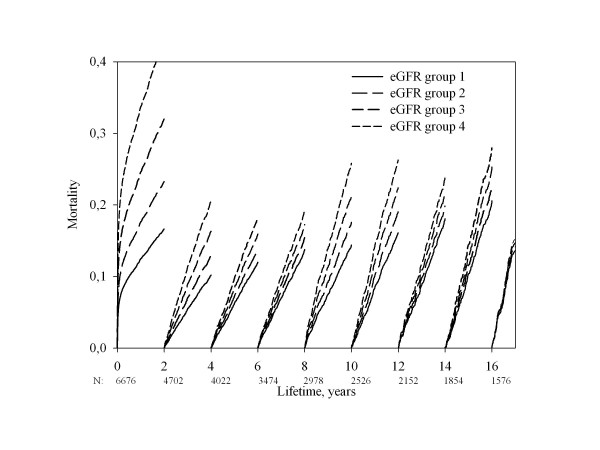
**Landmark analysis of the time dependent prognostic significance of renal function adjusted for age and gender**.

Renal function was inversely related to all-cause mortality (figure 1, unadjusted analysis). The mortality was 43,0% (eGFR group 1), 56,9% (eGFR group 2), 71,9% (eGFR group 3), 89,7% (eGFR group 4) at 10 years of follow-up and 57,7% (eGFR group 1), 71,3% (eGFR group 2), 83,6% (eGFR group 3), 95,4% (eGFR group 4) at 15 years of follow-up (p < 0.0001 for difference between the 4 eGFR groups). To clarify the importance of renal function as prognostic factor we performed a Landmark analysis illustrating survival stratified by eGFR group and adjusted for age and sex in 2 year intervals after the infarction (figure 2). Estimation of renal function has prognostic significance for up to 16 years following MI, even without adjustment for changing values of se-creatinine. Landmark analyses of 2-year periods shows that the statistic significance disappears after 12 years of follow-up, but hazard ratio is almost the same in the following years, so the lack of significance in this period is probably a result of lack of power. The hazard ratio is close to 1.00 only after 16 years of follow-up.

**Figure 3:**
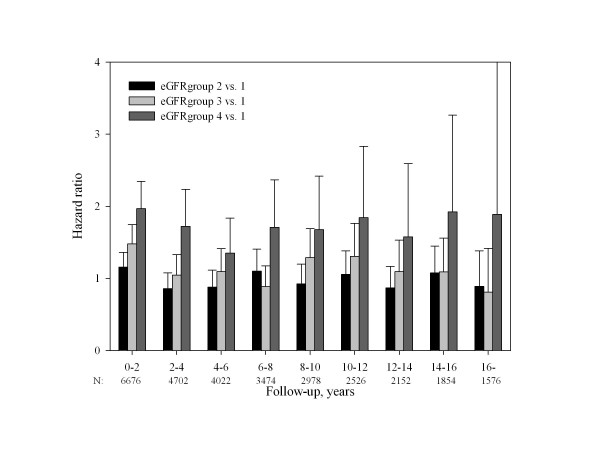
**Relative risk as a function of follow-up time in Cox proportional-hazard model 3 (all covariates)-eGFRgroups 2-4 with eGFRgroup 1 as reference**.

We constructed 3 Cox proportional-hazards models of total mortality with a stepwise addition of covariates (table 2). In the multivariable analyses we included demographic factors, comorbidities and information regarding thrombolytic therapy. We did not include information regarding complications because of the risk of immortal time bias. Since clinicians have varying thresholds for initiating medical therapy, we did not include information regarding the medical treatment at discharge in the multivariable analyses because this could make the interpretation of results very complex and difficult. Age was a significant prognostic factor throughout the follow-up in all models. When using eGFRgroup 1 (normal renal function) as reference in the model incorporating all covariates in the whole follow-up period, estimated GFR was a significant prognostic factor in eGFRgroups 3 (hazard ratio 1,19, CI 1,09-1,30) and 4 (hazard ratio 1,72, CI 1,56-1,91) but not in eGFRgroup 2 (hazard ratio 0,99, CI 0,91-1,07)(figure 3). Overall, there was a rise in hazard ratio with worsening renal function. eGFR was a prognostic factor for 12 years and s-creatinine for 10 years, with the exception of the follow-up period from 6-8 years where significance was only borderline. Restricting the analysis to eGFRgroup 4 (lowest eGFR group) and eGFR group 1 (normal eGFR), prognostic significance was observed in the entire period of follow-up with hazard ratio between 1,97 (CI 1,65-2,35) and 1,35 (CI 0,99-1,84). In all 2-year periods statistical significant impact of eGFR was found, with the exception of the last follow-up period, which was probably a result of a lack of statistical power. Also, the analysis of the 4-6 year period of follow-up showed borderline significance, again likely due to a lack of power. When we controlled the Cox-model incorporating all covariates for whether the patients received ACE-inhibitor, eGFR statistical significance was lost in the 4-6 years follow-up period, while the other results were not significantly changed (data not shown).

**Table 2:** Three proportional hazards models of mortality as a function of time with stepwise addition of variables

	0-2 years		2-4 years		4-6 years		6-8 years		8-10 years		10-12 years		12-14 years		14-16 years		16-years	
	N = 6676		N = 4702		N = 4022		N = 3474		N = 2978		N = 2526		N = 2152		N = 1854		N = 1576	
Variables	RR	CI*	RR	CI*	RR	CI*	RR	CI*	RR	CI*	RR	CI*	RR	CI*	RR	CI*	RR	CI*
**Model 1**																		
eGFRgroup**	1.77	1.69-1.85	1.57	1.46-1.70	1.45	1.33-1.58	1.43	1.30-1.56	1.53	1.39-1.68	1.42	1.27-1.58	1.40	1.24-1.58	1.36	1.20-1.55	1.28	1.04-1.57
Cregroup	2.09	1.96-2.23	1.95	1.71-2.22	1.89	1.61-2.21	1.58	1.30-1.91	1.68	1.36-2.06	1.51	1.17-1.95	1.55	1.14-2.10	0.91	0.63-1.33	1.57	0.93-2.63
Model 2***																		
Male gender	1.11	1.00-1.24	1.45	1.22-1.73	1.12	0.92-1.36	1.36	1.10-1.67	1.22	0.98-1.51	1.83	1.41-2.37	1.15	0.88-1.50	1.22	0.92-1.61	1.01	0.66-1.55
Age****	1.37	1.29-1.45	1.64	1.50-1.81	1.66	1.50-1.84	1.86	1.66-2.06	1.86	1.66-2.08	1.91	1.69-2.18	1.95	1.69-2.24	1.90	1.63-2.20	2.10	1.66-2.67
eGFRgroup**	1.27	1.20-1.34	1.21	1.11-1.32	1.12	1.01-1.23	1.10	0.99-1.22	1.20	1.07-1.34	1.19	1.05-1.35	1.11	0.96-1.29	1.13	0.97-1.31	1.01	0.79-1.29
Cregroup	1.34	1.24-1.46	1.30	1.12-1.51	1.47	1.24-1.74	1.10	0.90-1.36	1.30	1.05-1.63	1.25	0.96-1.62	1.28	0.93-1.76	0.76	0.52-1.11	1.35	0.79-2.32
eGFRgroup 2 vs. 1	1.15	0.98-1.36	0.86	0.68-1.08	0.88	0.69-1.12	1.10	0.86-1.41	0.92	0.71-1.20	1.06	0.81-1.38	0.87	0.64-1.17	1.08	0.80-1.45	0.89	0.57-1.38
eGFRgroup 3 vs. 1	1.48	1.26-1.75	1.04	0.82-1.33	1.10	0.85-1.41	0.89	0.67-1.18	1.29	0.98-1.69	1.30	0.96-1.76	1.10	0.78-1.53	1.09	0.76-1.56	0.81	0.46-1.42
eGFRgroup 4 vs. 1	1.97	1.65-2.35	1.72	1.32-2.24	1.35	0.99-1.84	1.71	1.23-2.37	1.68	1.16-2.42	1.84	1.20-2.83	1.58	0.96-2.59	1.92	1.13-3.26	1.89	0.78-4.55

In all models either cregroup or eGFR were included in the model.

*95% confidence interval

**Hazard ratio associated with 1 eGFR group improvement

***Other covariates in model 2: Body mass index, previous AMI, angina pectoris, congestive heart failure, diabetes mellitus, wall motion index, systemic hypertension, thrombolytic therapy.

****Hazard ratio associated with an increase in age of 10 years.

In order to examine whether the prognostic significance of renal function was different in patients revascularized with thrombolysis, we performed an interaction analysis between renal function and trombolysis. We found a significant interaction between creatinine groups and thrombolysis with a p-value of 0.007. The overall hazard ratio was 1.374 (CI 1.289-1.464) with a hazard ratio in the group not treated with thrombolysis of 1.367 (CI 1.281-1.460) and 1.149 (CI 1.030-1.281) in the group treated with trombolysis. Similarly, an interaction was seen between eGFRgroup and thrombolysis with a p-value of 0.001. The overall hazard ratio was 1.242 (CI 1.194-1.292), with a hazard ratio of 1.235 (CI 1.186-1.287) in the group not treated with thrombolysis and 1.128 (CI 1.068-1.191) in the group treated with thrombolysis. In conclusion, the prognostic significance was greatest in the group not treated with thrombolysis, but as can be seen from the numbers, the interaction was not due to a large difference in hazard ratio associated with a decrease in renal function.

## Discussion

We found that renal function measured during the index hospitalization was an independent significant prognostic factor more than 10 years after MI.

Ideally, the evaluation of renal function should be performed by direct GFR measurement, but this approach is often limited by practical considerations. Several studies have used se-creatinine instead of GFR to evaluate renal function. However, the association between se-creatinine and GFR is nonlinear, because it varies with age, gender, ethnicity and lean body mass, which limits the value of serum creatinine level as a marker of renal function [18-20]. As a result, the National Kidney Foundation uses GFR, not serum creatinine, to define chronic kidney disease [17,19]. Of the several reliable formulas incorporating clinical variables, we used the MDRD formula, which has previously been used in 2 large studies [11,18]. However, a study by Szummers showed that Cockcroft-Gault formula was better than the MDRD equation at predicting mortality after a MI. This was mainly explained by differences in the coefficients and variables included in the eGFR equations, and less to differences in various subgroups of patients [21]. The limited value of se-creatinine measurement was illustrated in our study by modest differences in se-creatinine between eGFR groups 1-3, despite large differences in eGFR which were also associated with differences in all-cause mortality. Nevertheless, despite its shorter persistence, se-creatinine level was as strong a prognostic factor as eGFR. The hazard ratios for creatinine groups were slightly higher than for eGFR groups in some 2-year periods of follow-up, but also more imprecise.

The observed increase in risk of all-cause mortality with decreasing eGFR is likely explained by factors associated with renal decline, for example albuminuria, proteinuria, homocysteinemia [1,2,22]. Elevated uric acid levels and traditional risk factors for coronary heart disease are prevalent in patients with chronic kidney disease [2]. Hypertension may cause or worsen renal disease and is a significant cause of the accelerated development of cardiovascular disease [23]. Indeed, patients with impaired renal function have more severe and diffuse coronary heart disease [24]. A vicious circle often exists because the frequency of coronary risk factors increases with decreasing renal function. In our study, impaired renal function was associated with a higher frequency of hypertension, diabetes, angina pectoris and previous MI. Impaired renal function was also associated with higher age and was more common among women, both factors which reduce GFR estimated by the MDRD formula. We found that age was a significant risk factor for all-cause mortality throughout the length of follow-up, which is in agreement with previous studies [1,22].

Many patients with decreased renal function are treated less aggressively with therapeutic interventions and risk factor modification, a finding documented to parallel a worsening in renal function [25,26]. Patients with end-stage renal disease, whom are known to be at very high risk for cardiovascular events, are also treated less aggressively with prognosis-improving cardiovascular medications [27,28]. The reason often is a fear of worsening renal function and of drug-induced toxic effects as a result of low clearance. In our study, fewer patients received thrombolytic therapy when renal function decreased. This could be a result of less aggressive treatment of patients with renal dysfunction, but it is probably also explained by the age-difference between patients with decreased and normal renal function, since the se-creatinine value is usually not known at the time of initiation of thrombolytic therapy.

The management of patients with decreased renal function is difficult. On one hand, patients with renal impairment can safely obtain the same benefits of cardiovascular medications and invasive procedures when appropriately monitored [29-34]. On the other hand, many of the large landmark cardiovascular trials have excluded patients with renal function under a certain limit.

It has been documented that in patients with diabetes, the prevalence of renal dysfunction is higher, but the effect of renal function on cardiovascular risk is largely independent of diabetes status. Thus, a similar risk relation between renal function and CV risk exists in patients with and without DM [35].

Several recent papers have examined the prognostic role of renal function in AMI patients. One study examined the prognostic significance of an acute worsening of renal function among patients hospitalised for MI surviving to hospital discharge. With a follow-up of at least 4 years, worsening renal function was independently associated with diabetes, left ventricular systolic dysfunction and a history of chronic kidney disease. After adjustment for factors associated with worsening renal function and long-term mortality, worsening renal function was independently associated with a higher risk of death [36]. In another study, the prognostic significance of chronic kidney disease and acute kidney injury on acute coronary syndrome was reviewed. This study stressed the importance of early measurement and monitoring of renal function, which is probably standard of care in most institutions [37]. The significance of creatinine clearance at the time of hospital admission as a predictor of in-hospital adverse events and mortality was examined using data from the global registry of acute coronary events (GRACE) comprising 11 774 patients hospitalised with ACS. The results showed that in comparison with patients with normal or minimally impaired renal function, patients with moderate renal dysfunction were twice as likely to die and those with severe renal dysfunction almost four times more likely to die after adjustment for other potentially confounding variables. The risk of major bleeding episodes increased as renal function worsened [38]. Data from GRACE also showed that initial serum creatinine concentration was among the nine factors that independently predicted death and the combined end point of death and myocardial infarction in the period from admission to six months after discharge [39]. An observational study of 57477 consecutive MI patients showed that declining GFR estimated by the MDRD formula was associated with an increased rate of complications and a higher rate of in-hospital mortality [40].

The population described here was recruited from 27 Danish hospitals with regional patient uptake and can be considered representative in a western industrialized country of patients admitted alive with MI. With declining renal function, patients in our study were more often women, were older and had a greater frequency of comorbidities. This is in accordance with previous studies [38,40]. We have shown that reduced renal function is a long-term independent predictor of all-cause mortality in a graded manner. Previous studies have documented adverse outcomes at 30 and 180 days in patients with reduced renal function of MI [3,24,41-43]. The novelty of our results is the description of the prognostic effect of renal function in 2-year intervals with a very long follow-up period. This makes it possible to document the loss of prognostic significance of a risk factor in the very long-term.

The relationship between renal function and cardiovascular outcomes has previously been examined in several large studies: the Studies of Left Ventricular Dysfunction (SOLVD) trial, Trandolapril Cardiac Evaluation (TRACE) trial, Survival and Ventricular Enlargement (SAVE) trial and 2 analyses of the Valsartan in Acute Myocardial Infarction Trial (VALIANT) [4,11,35,44,45]. The SOLVD and SAVE studies and the analyses from VALIANT used the MDRD formula to estimate renal function. All these studies found that reduced renal function was independently associated with an increased risk of death and cardiovascular outcomes. Our study also comprises a large cohort but with a broader spectrum of renal dysfunction, because all patients in the TRACE register was included irrespective of se-creatinine level [11,35]. Together with the inclusion of consecutive patients we believe this makes the population studied here is more representative of daily clinical practice. Moreover, our cohort was followed for up to 17 years, far longer than any other study on this subject. In comparison, the analysis from VALIANT examined only high-risk patients with clinical or radiological signs of heart failure and/or left ventricular systolic dysfunction and the follow-up period was limited to a median of 24.7 months.

## Limitations

Our study has several limitations that should be acknowledged. The data do not allow us to evaluate the effect of duration or changes in renal dysfunction. Contrast-medium-induced nephropathy related to coronary angiography cannot be evaluated. The MDRD equation has limitations, because serum creatinine is influenced by non-renal factors. The baseline-estimated GFR can be affected by urinary excretion of protein, which we did not measure. Our study focuses solely on mortality, with no information on the effect of renal dysfunction on nonfatal outcomes. We do not have information regarding how many patients underwent coronary angiography or CABG during hospital stay or later. This treatment was not routine at the time of the trial (1992-1994). As a result, we cannot evaluate whether the frequency of invasive treatment differ in the eGFRgroups. It has previously been shown that early invasive therapy defined as revascularization within 14 days of admission was associated with greater 1-year survival in patients with non-ST-elevation myocardial infarction and mild-to-moderate renal insufficiency, but the benefit declined with lower renal function [46]. It might be argued that the long-term prognostic information is of lesser interest for renal function, which is frequently updated be creatinine measurement, than for other risk factors, for example systolic function by echocardiography, which is updated more rarely.

## Conclusions

Renal function is a strong and independent long-term prognostic factor the first 10-12 years following a MI. Estimating GFR provides a more precise estimate of renal function than se-creatinine and carries stronger prognostic significance. We expect that the prevalence of renal dysfunction among patients with MI will increase as a result of an ageing population with an accumulation of risk factors, including hypertension and diabetes. Consequently, the relevance of the findings of the present study will be relevant also in the future.

## Competing interests

The authors declare that they have no competing interests.

## Authors' contributions

TK was responsible for all stages of the study with the exception of data collection and follow-up: Analysis of data, analysis of the literature, and preparation of the manuscript. GG supervised the entire study, participated in follow-up, analysis and interpretation of data, and revised the manuscript. LK supervised the entire study, participated in follow-up, analysis and interpretation of data, and revised the manuscript. FG aided in analysis and interpretation of data and revision of the manuscript. MS aided in analysis and interpretation of data and revision of the manuscript. CT-P conceived the idea for the study, supervised the study, collected data, analyzed data and revised the manuscript. All authors have seen and approved the manuscript.

## Pre-publication history

The pre-publication history for this paper can be accessed here:

http://www.biomedcentral.com/1471-2261/11/37/prepub
